# Nicotinamide Adenine Nucleotide—The Fountain of Youth to Prevent Oocyte Aging?

**DOI:** 10.3390/cells10092441

**Published:** 2021-09-16

**Authors:** Paweł Kordowitzki, Wing-Hong Jonathan Ho, Dave R. Listijono

**Affiliations:** 1 Institute of Animal Reproduction and Food Research of Polish Academy of Sciences, Tuwima Street 10, 10-243 Olsztyn, Poland; 2 Faculty of Biology and Veterinary Medicine, Nicolaus Copernicus University, Gagarina Street 7, 87-100 Torun, Poland; 3 The Kinghorn Cancer Centre, Cancer Division, Garvan Institute of Medical Research, 384 Victoria Street, Darlinghurst, NSW 2010, Australia; w.ho@garvan.au; 4 St. Vincent’s Clinical School, St. Vincent’s Hospital, 390 Victoria Street, Darlinghurst, NSW 2010, Australia; 5 IVF Australia, Level 1/33 York Street, Sydney, NSW 2000, Australia; d.listijono@gmail.com

**Keywords:** nicotinamide adenine nucleotide, aging, oocyte, NAD+, sirtuins

## Abstract

According to the U.S. Special Operations Command (SOCOM), new clinical trials of an anti-aging oral treatment using nicotinamide adenine nucleotide are planned for 2022. All over the globe, the discovery of the fountain of youth is still a great goal to reach, not only among aging researchers, since people desire to stay longer healthy and feel young when reaching old age. Since the 1960s, women delaying pregnancy to pursue higher educational levels and a career path has contributed to drastically diminished overall female fertility rates (e.g., number of born offspring/woman). Consequently, a growing number of advanced-aged women depend on assisted reproductive technologies (ART) to become pregnant. In 2019, the Society for Assisted Reproductive Technology reported 293,672 cycles for oocyte retrieval. This change of demographics influenced women’s age of having their first child, which has increased significantly. However, their reproductive tract shows hallmarks of aging very early in life without an effective preventive treatment. Therefore, we will present whether NAD+ could help to prevent oocyte aging.

## 1. Introduction

**Figure 1:**
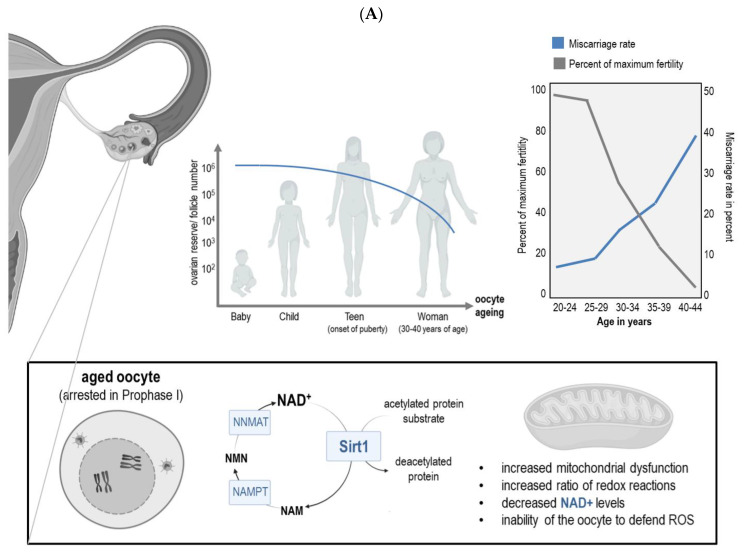

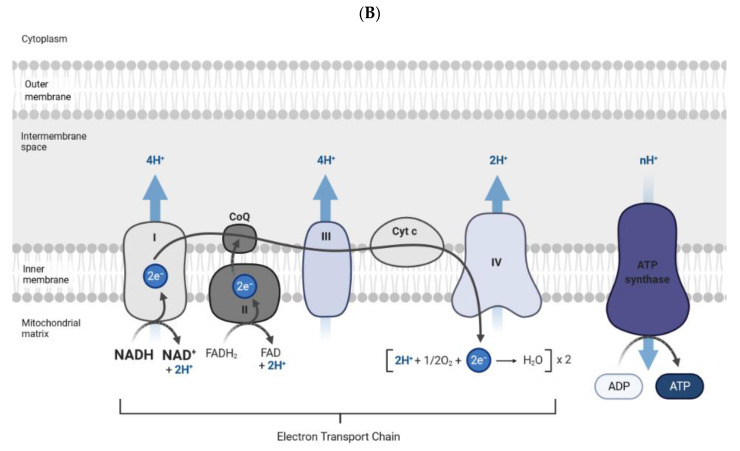
Impact of aging on NAD+ levels and oocyte number, and oocyte quality. (**A**) The blue line in the graph shows the gradual decline of the follicular pool, which is more enhanced after the age of 35 years in women. The oocytes in theses follicles are arrested in Prophase of the first meiotic division, and with advancing maternal age, oocytes’ mitochondria lose their function more and more. In consequence, NAD is less available, the oocyte has reduced quality and difficulties to defend against ROS. Sirt1 uses NAD+ as co-factor during the deacetylation of acetylated proteins. During this process nicotinamide is produced, which is then recycled back into NAD+. (**B**) Scheme showing the involvement of NADH/NAD+ in the electron transport chain which transports electrons along the mitochondrial membrane through the single complexes (I–IV). Abbreviations: ADP = adenosine diphosphate, ATP = adenosine triphosphate, CoQ = co-enzyme Q, Cyt c = cytochrome c, NAM = nicotinamide, NMN = nicotinamide mononucleotide, NAMPT= nicotinamide phosphoribosyl-transferase, NNMAT = nicotinamide mononucleotide adenylyl-transferase.

Many reports have highlighted that mitochondria are the powerhouses in the ovum, the largest cell in the human body [1]. The main process of energy production in oocytes is oxidative phosphorylation, a biochemical reducing and oxidizing (redox) process during which reactive oxygen species (ROS) are generated as a by-product. For the latter mentioned process, the electron transport chain plays a fundamental role (Figure 1). Previous studies provided strong evidence that the oocyte’s redox level determines its developmental competence. In numerous cellular redox reactions, the co-factor nicotinamide adenine nucleotide (NAD) plays a very crucial role, and NAD levels have been reported to decrease with age (Figure 1), which has been linked to aging-related mitochondrial dysfunction [1]. Three main strategies to counteract the aging-related decline of NAD could represent the possible fountain of youth, not only for the oocyte, such as avoiding/minimizing NAD degradation, supplementing NAD precursors, and activating NAD biosynthetic enzymes [1,2]. The “free radical theory of aging” [3] implies a reduced potential of oocytes to counteract ROS. Interestingly, evidence suggests that the protein Sirt1 not only relies on NAD+ as a cofactor (Figure 1), but is also able to slow down age-related decline in oocyte quality [4]. Recent DNA-methylation and epigenetic clock studies on oocytes revealed that some CpG probes downstream and only one probe upstream of SIRT1 were hypomethylated with advancing maternal age [5]. Cellular senescence was proposed to be the central biological pathway for several diseases related to aging [6]. Senescent cells show specific characteristics such as the arrest of growth/proliferation, and a senescence-associated secretory phenotype, in which, among others, cytokines and chemokines are secreted, which then provoke tissue remodeling and inflammation [7]. Importantly, typical for senescent cells are the glycolytic and mitochondrial oxidative phosphorylation activities, and there is an increased NAD+ metabolism present due to elevated expression of nicotinamide phosphoribosyl-transferase (NAMPT) expression (Figure 1A) [7]. It has been reported that the lack of NAMPT diminishes the availability of NAD+ by which cellular senescence is promoted [8]. A recent study provided evidence that senescent cells promote the decrease of NAD+ during the process of aging [9]. These findings suggest that NAD+ is an important determinant factor in cellular senescence development but whether its expression rule is senescence-associated secretory phenotype promoter or suppressor maybe cell-type specific. Besides NAD+, several other so-called senolytic drugs, either naturally occurring compounds such as Quercetin and Curcumin, or synthetic molecules, have been reported to have an anti-senescence effect [10]. Interestingly, a recent study showed that Quercetin was able to enhance the in vitro maturation of aged murine oocytes [11]. Similar to the NAD+ anti-senescence effect, the polyphenolic compound procyanidin C1, another senolytic agent and possible competitor to NAD+, has recently been shown to inhibit the senescence-associated secretory phenotype, and also promoted the maturation success of porcine oocytes when used in vitro [12].

This commentary aims to explain if and how NAD could be used as a potential therapeutic target to protect oocytes from aging, or in other words, whether NAD could be the fountain of youth to extend female reproductive lifespan. 

## 2. Nicotinamide Adenine Dinucleotide as a Potential Therapeutic Target to Preserve Oocyte Quality from Aging 

The preservation of oocyte quality against aging is one of the most challenging tasks in female reproductive research. According to the increasing maternal age, a therapeutic intervention to target this issue is urgently needed. Recently, there has been increased attention on studying the benefit of nicotinamide dinucleotide (NAD+) on human health; therefore, the U.S. Special Operations Command has plans for new clinical trials of an anti-aging oral treatment for 2022. In a study where mice were used as an animal model, it was shown that boosting NAD+ levels by the long-term administration of its precursor, nicotinamide mononucleotide (NMN), through non-aggressive drinking water, not only prevented the reduction of ovarian reserve but also restored oocyte quality and fertility function in aged mice [13]. In particular, maintaining the expression of NAD+ of aged oocytes in the level comparable in young oocytes prevented defeated spindle integrity, reduced oxidative stress, and improved live birth rate [13]. 

An increasing number of studies showed the interest in understanding how NAD+ protects female fertility in aging. One possible explanation for the latter mentioned question could be found in the link between NAD and sirtuins. Sirtuins belong to a family of NAD-dependent deacetylases with seven members (SIRT1-7), and some of them have been found to have a regulatory role in the female reproductive system [14,15,16,17]. Preventing the decline of sirtuins over time has been proven to have a protective role in female fertility in multiple aspects including a better regulation of oocyte development and maintaining spindle integrity. For instance, among others, SIRT1 has been suggested to mediate the beneficial effects of mammalian target of rapamycin (mTOR) suppression on ovarian lifespan via sustaining the primordial follicle pool in rat ovaries [18]. Additionally, SIRT6 knockout oocytes also displayed increased spindle defects, chromosome misalignment and higher aneuploidy rates—the effects which are likely due to SIRT6-knockdown-induced hyperacetylation of H4K16 [19,20]. These findings strongly support the protective role of sirtuins and indicate that the supplementation of NMN and/or NAD could be a potential treatment to preserve ovarian reserve and oocyte quality from advanced age females. 

## 3. Conclusions

Importantly, it needs to be elucidated why oocytes age so early, years before other age-related symptoms occur, and how NAD could be used as an anti-aging treatment in women to develop future strategies for extending the oocyte’s lifespan and quality at an adequate maternal age. The new clinical trials announced by the SOCOM could help to understand what levels of NAD+ are present in healthy aging and age-related diseases. Oocytes generated from animals could represent an interesting model to study how NAD+ contributes to delay, prevent, or maybe even reverse hallmarks of aging. The advances and improvement of assisted reproductive technologies and biotechnologies in the past thirty years has made it possible to create models to study the interplay between single pathways and processes in oocytes during meiosis. Taken together, our commentary aimed to stimulate new research ideas on this interesting topic, which could aid in the design of novel studies and animal models.

## References

[1] Van der Reest J., Nardini Cecchino G., Haigis M.C., Kordowitzki P. (2021). Mitochondria: Their relevance during oocyte ageing. Ageing Res. Rev..

[2] Rajman L., Chwalek K., Sinclair D.A. (2018). Therapeutic potential of NAD-boosting molecules: The in vivo evidence. Cell Metab..

[3] Tarín J.J. (1995). Aetiology of age-associated aneuploidy: A mechanism based on the ‘free radical theory of ageing’. Hum. Reprod..

[4] Iljas J.D., Wei Z., Homer H.A. (2020). Sirt1 sustains female fertility by slowing age-related decline in oocyte quality required for post-fertilization embryo development. Aging Cell.

[5] Kordowitzki P., Haghani A., Zoller J.A., Li C.Z., Raj K., Spangler M.L., Horvath S. (2021). Epigenetic clock and methylation study of oocytes from a bovine model of reproductive aging. Aging Cell.

[6] Boccardi V., Mecocci P. (2021). Senotherapeutics: Targeting senescent cells for the main age-related diseases. Mech. Ageing Dev..

[7] Schug Z., Tang H.Y., Speicher D.W., David G., Zhang R. (2019). NAD^+^ metabolism governs the proinflammatory senescence-associated secretome. Nat. Cell. Biol..

[8] Jadeja R.N., Powell F.L., Jones M.A., Fuller J., Joseph E., Thounaojam M.C., Bartoli M., Martin P.M. (2018). Loss of NAMPT in aging retinal pigment epithelium reduces NAD^+^ availability and promotes cellular senescence. Aging.

[9] Covarrubias A.J., Kale A., Perrone R., Lopez-Dominguez J.A., Pisco A.O., Kasler H.G., Schmidt M.S., Heckenbach I., Kwok R., Wiley C.D. (2020). Senescent cells promote tissue NAD^+^ decline during ageing via the activation of CD38^+^ macrophages. Nat. Metab..

[10] Lagoumtzi S.M., Chondrogianni N. (2021). Senolytics and senomorphics: Natural and synthetic therapeutics in the treatment of aging and chronic diseases. Free Radic. Biol. Med..

[11] Cao Y., Zhao H., Wang Z., Zhang C., Bian Y., Liu X., Zhang C., Zhang X., Zhao Y. (2020). Quercetin promotes in vitro maturation of oocytes from humans and aged mice. Cell Death Dis..

[12] Xu Q., Fu Q., Li Z., Liu H., Wang Y., Lin X., He R., Zhang X., Campisi J., Kirkland J.L. (2021). Procyanidin C1 is a natural agent with senolytic activity against aging and age-related diseases. bioRxiv.

[13] Bertoldo M.J., Listijono D.R., Ho W.J., Riepsamen A.H., Goss D.M., Richani D., Jin X.L., Mahbub S., Campbell J.M., Habibalahi A. (2020). Nad(+) repletion rescues female fertility during reproductive aging. Cell Rep..

[14] Bonkowski M.S., Sinclair D.A. (2016). Slowing ageing by design: The rise of Nad(+) and sirtuin-activating compounds. Nat. Rev. Mol. Cell Biol..

[15] Chang H.C., Guarente L. (2014). Sirt1 and other sirtuins in metabolism. Trends Endocrinol. Metab..

[16] Haigis M.C., Sinclair D.A. (2010). Mammalian sirtuins: Biological insights and disease relevance. Annu. Rev. Pathol..

[17] Tatone C., di Emidio G., Vitti M., di Carlo M., Santini S., D’Alessandro A.M., Falone S., Amicarelli F. (2014). Sirtuin functions in female fertility: Possible role in oxidative stress and aging. Oxid. Med. Cell Longev..

[18] Zhang X.M., Li L., Xu J.J., Wang N., Liu W.J., Lin X.H., Fu Y.C., Luo L.L. (2013). Rapamycin preserves the follicle pool reserve and prolongs the ovarian lifespan of female rats via modulating mtor activation and sirtuin expression. Gene.

[19] Longsen H., Ge J., Zhang L., Ma R., Hou X., Li B., Moley K., Wang Q. (2015). Sirt6 depletion causes spindle defects and chromosome misalignment during meiosis of mouse oocyte. Sci. Rep..

[20] Ma P., Schultz R.M. (2013). Histone deacetylase 2 (Hdac2) regulates chromosome segregation and kinetochore function via H4k16 deacetylation during oocyte maturation in mouse. PLoS Genet..

